# Effectiveness of community and school-based sanitation interventions in improving latrine coverage: a systematic review and meta-analysis of randomized controlled interventions

**DOI:** 10.1186/s12199-021-00934-4

**Published:** 2021-02-24

**Authors:** Satoshi Igaki, Nguyen Tran Minh Duc, Nguyen Hai Nam, Tran Thi Tuyet Nga, Parshal Bhandari, Amr Elhamamsy, Caroline Ibrahim Lotify, Mostafa Elsayed Hewalla, Gehad Mohamed Tawfik, Peterson Gitonga Mathenge, Masahiro Hashizume, Nguyen Tien Huy

**Affiliations:** 1 School of Tropical Medicine and Global Health, Nagasaki University, Nagasaki, Japan; 2 Online Research Club, http://www.onlineresearchclub.org/; 3 University of Medicine and Pharmacy, Ho Chi Minh City, 70000 Vietnam; 4 Department of General Surgery, University of Medicine and Pharmacy at Ho Chi Minh City, Ho Chi Minh City, 70000 Vietnam; 5 Faculty of Public Health, University of Medicine and Pharmacy at Ho Chi Minh City, Ho Chi Minh City, Vietnam; 6 Department of Anesthesiology and Intensive Care, Post Graduate Medical Institute, Lahore General Hospital, Lahore, Pakistan; 7 Faculty of Pharmacy, Tanta University, Tanta, Egypt; 8 Faculty of Pharmacy, Assiut University, Assiut, Egypt; 9 Faculty of Medicine, Alexandria University, Alexandria, Egypt; 10 Faculty of Medicine, Ain Shams University, Cairo, Egypt; 11 Leading Program, Graduate School of Biomedical Sciences, Nagasaki University, Nagasaki, Japan; 12 Department of Immunogenetics, Institute of Tropical Medicine (NEKKEN), Nagasaki University, Nagasaki, Japan; 13 Department of Pediatric Infectious Diseases, Institute of Tropical Medicine (NEKKEN), Nagasaki University, Nagasaki, Japan; 14 Institute of Research and Development, Duy Tan University, Da Nang, 550000 Vietnam; 15 Department of Clinical Product Development, Institute of Tropical Medicine (NEKKEN), School of Tropical Medicine and Global Health, Nagasaki University, Nagasaki, Japan

**Keywords:** Sanitation, Latrine, Meta-analysis, Systematic review

## Abstract

**Introduction:**

Approximately 1000 children die each year due to preventable water and sanitation-related diarrheal diseases. Six in 10 people lacked access to safely managed sanitation facilities in 2015. Numerous community- and school-based approaches have been implemented to eradicate open defecation practices, promote latrine ownership, improve situation sanitation, and reduce waterborne disease.

**Objective:**

Given that current evidence for sanitation interventions seem promising, the aim of this study was to systematically summarize existing research on the effectiveness of community- and school-based randomized controlled sanitation intervention in improving (1) free open defecation (safe feces disposal), (2) latrine usage, (3) latrine coverage or access, and (4) improved latrine coverage or access.

**Methods:**

Eight electronic databases were searched: PubMed, Scopus, WHO Global Health Library (GHL), Virtual Health Library (VHL), POPLINE, Web of Science, Cochrane, and Google Scholar up to 26 April 2019. Original randomized clinical trials addressing community-based or school-based intervention that reported feces disposal and latrine coverage were deemed eligible. More than two researchers independently contributed to screening of papers, data extraction, and bias assessment. We conducted a meta-analysis by random-effects model. The risk of bias was assessed by the Cochrane risk of bias tool.

**Results:**

Eighteen papers that matched all criteria and 16 studies were included in the final meta-analysis. Compared to the control, the sanitation intervention significantly increased safe feces disposal (OR 2.19, 95% CI 1.51–3.19, *p* < 0.05, *I*^2^ = 97.28), latrine usage (OR 3.72, 95% CI 1.71–8.11, *p* < 0.05, *I*^2^ = 91.52), latrine coverage or access (OR 3.95, 95% CI 2.08–7.50, *p* < 0.05, *I*^2^ = 99.07), and improved latrine coverage or access (OR 3.68, 95% CI 1.52–8.91, *p* < 0.05, *I*^2^ = 99.11). A combination of education and latrine construction was more effective compared to educational intervention alone.

**Conclusion:**

Our study showed strong evidence for both community- and school-based sanitation interventions as effective for the safe disposal of human excreta. The finding suggests major implications for health policy and design of future intervention in developing countries.

**Supplementary Information:**

The online version contains supplementary material available at 10.1186/s12199-021-00934-4.

## Introduction

According to the World Health Organization (WHO) report (2017), diarrheal disease is the second leading cause of death worldwide in childhood under 5 years of age. Diarrheal diseases significantly impact underdeveloped countries, which often faces insufficient safe water supplies, poor hygiene conditions, and lower sanitation levels. Diarrheal diseases are associated with infection by and dissemination of various pathogens with significant adverse public health scenarios. However, a significant amount of diarrheal disease can be prevented [1–3].

The United Nations (UN) set the Sustainable Development Goals (SDGs) in 2015, which succeeded in the Millennium Development Goals (MDGs). The SDGs were designed to ensure availability and sustainable management of water access, sanitation and addressed sanitation and hygiene topics in Goal 6. According to the World Health Organization (WHO) and the United Nations Children’s Fund (UNICEF) Joint Monitoring Program for Water Supply, Sanitation, and Hygiene (JMP) report (2017), 6 in 10 people lacked access to safely managed sanitation facilities in 2015. Moreover, there was a decrease in the number of people who practiced open defecation from 1229 million to 892 million with an average decline of 22 million people per year. These people have received limited education on sanitation and hygiene conditions [4]. Open defecation is a persistent issue as it increases fecal exposure and leads to unfavorable health outcomes. Various approaches have been taken to reduce open defecation. For example, the community-led total sanitation (CLTS) approach developed by Drs. Kamal Kar and Robert Chambers [5] was launched in Bangladesh in 2000 and has been implemented in many developing countries. A total sanitation campaign (TSC) was initiated in 1999–2012 as an attempt by the Indian government to eradicate open defecation practices and promote latrine ownership through nationwide hardware and software support. The average increase in latrine coverage was 27% (95% confidence interval 14–39) [6]. Another major approach was the water, sanitation, and hygiene (WASH) intervention, designed to supply sufficiently safe water and affordable hygiene stations to improve the level of sanitation and prevent waterborne diseases. Wolf et al. showed a large reduction of diarrheal disease risk through interventions through improved drinking water, sanitation, and hygiene [7].

Two systematic reviews have evaluated the effects of sanitation interventions on latrine coverage and use, and study the quality of CLTS [6, 8]. Garn et al. [6] described latrine coverage and latrine use by meta-analysis: overall, the average increase in latrine coverage in communities undergoing sanitation intervention was 14% (95% CI 10–19), compared with control community. Significant heterogeneity was observed across studies (*I*^2^ = 94.2%, *p* < 0.001) presumably arising from different interventions and various study designs on latrine coverage. Venkataramanan et al. [8] assigned quality appraisal scores according to literature type and study design, a summarized quantitative evaluation, indicators of progress, and outcomes measured in CLTS programs, and assessed factors that facilitated or constrained implementation by stage of CLTS. Garn et al. [6] analyzed sanitation structure and design characteristics and their association with latrine use. They also assessed the impact of sanitation intervention on household latrine coverage and/or use through a meta-analysis of before-and-after, randomized, and non-randomized controlled trials.

This systematic review is done to show the impact of community- and school-based sanitation interventions in improving latrine use and coverage. Moreover, it also relays information about how different interventions help with safe fecal disposal and reduce diarrheal disease incidence/prevalence in any community. Our study only included randomized control trials to eliminate the risk of selection bias and to show the real difference between intervention and control groups regarding the outcome, which made it superior to other studies.

## Methods

### Search strategy

This study was conducted based on the Preferred Reporting Items for Systematic Review and Meta-Analysis (PRISMA) guideline [9] as presented in the PRISMA checklist (Supplemental Table 1). Our protocol was registered and published with PROSPERO (CRD42019130120). In February 2019, we systematically searched with no restriction on language or data for potential studies via eight electronic databases: PubMed, Scopus, WHO Global Health Library (GHL), Virtual Health Library (VHL), POPLINE, Web of Science, Cochrane, and Google Scholar. Our primary outcome was latrine coverage among the community. The following information was extracted from each study; the prevalence of safe feces disposal, latrine usage, and latrine coverage or access, diarrhea prevalence, study design, methods, and types of intervention programs. A comprehensive search term was represented in Supplemental Table 2. Manual search was also performed by screening the references of the included studies, the related studies suggested by PubMed, Google Scholar in the first page, and the reference of reviews relevant to this theme. The last manual search was performed on 20 June 2019.

### Selection criteria and title/abstract screening

We included the studies that met the following inclusion criteria: (1) community-based or school-based randomized controlled intervention on the sanitation and/or hygiene; (2) reported feces disposal; (3) studied latrine coverage and/or usage; (4) no restrictions on race, ethnicity, age, sex, language, geographical area, or place. The exclusion criteria were (1) in vitro or animal model studies; (2) case series, case reports, letters, editorials, theses, review protocols, conference abstracts, or book chapters; (3) data cannot be extracted or duplicated studies; (4) no full text available. The search results were imported from eight databases into Endnote X9 (Thompson Reuter, USA) for deletion of duplicates. Titles and abstracts were independently screened by at least two reviewers for potential relevance using predetermined criteria. Full-text screening was conducted when screening titles and abstracts were insufficient to decide. We intended to translate non-English articles; however, all included studies were in English. The full texts of the included study papers were downloaded through the Library of Nagasaki University. Subsequently, full-text screening was used to select relevant articles for data extraction. Any conflicts were resolved through discussion and obtained a consensus among reviewers and the reviewing supervisor.

### Data extraction

The data extracted from articles included the name of the first author, year of publication, name of journal, study country, intervention description, study design, study purpose, sample size, baseline and end line timing, socio-demographic characteristics of intervention and control groups, latrine coverage/access, latrine usage, sanitation outcomes, supportive outcomes, study limitations, and other comparative data. Data that were not available were filled in as “N/A,” which means “not applicable.” At least two people independently conducted data extraction to prevent missing data and mistakes. Any disagreement was resolved by discussion and consensus to avoid data extraction error. In the case of unreliable or missing data, an email was sent twice to the corresponding authors requesting the missing information.

### Statistical methods

Statistical analysis was carried out using Comprehensive Meta-Analysis Software (version 3.0, Biostat, Inc., USA). Meta-analysis was applied for any outcome when reported by two studies or more studies. A fixed-effects model was used when there was no evidence of heterogeneity between studies. Otherwise, a random-effects model was used. Significant statistical heterogeneity was considered when the *I*^2^ test > 50% or *p* value from *χ*2 tests [10]. To statistically evaluate the presence of publication bias, we used Egger’s regression test [11] and Begg’s funnel plot [12]. Publication bias was significantly measured when the *p* value was less than 0.1. If publication bias was found, the trim and fill method of Duvall and Tweedie was used to add studies and enhance the symmetry [13]. We also conducted sensitivity analyses to evaluate the effect of each study on the association. The adjusted pooled OR and its 95% confidence interval (95% CI) was computed, and statistical significance was considered if the *p* value was < 0.05 (two-tailed test) or 95% CI did not overlap with the original one.

### Quality assessment

Three reviewers independently assessed the included studies for risk of bias and methodological quality by using Cochrane Collaboration’s tool for RCTs [14]. This tool contains seven components: randomization, allocation concealment, blinding of subjects, blinding of outcome assessors, reporting of incomplete outcome data, selective outcome reporting, and other potential sources of bias. Each of the seven categories was judged by using one of three markings as high risk, low risk, or unclear risk of bias. If any disagreement happened, it was solved by reviewers and a supervisor, and a consensus was reached.

## Results

**Fig. 1:**
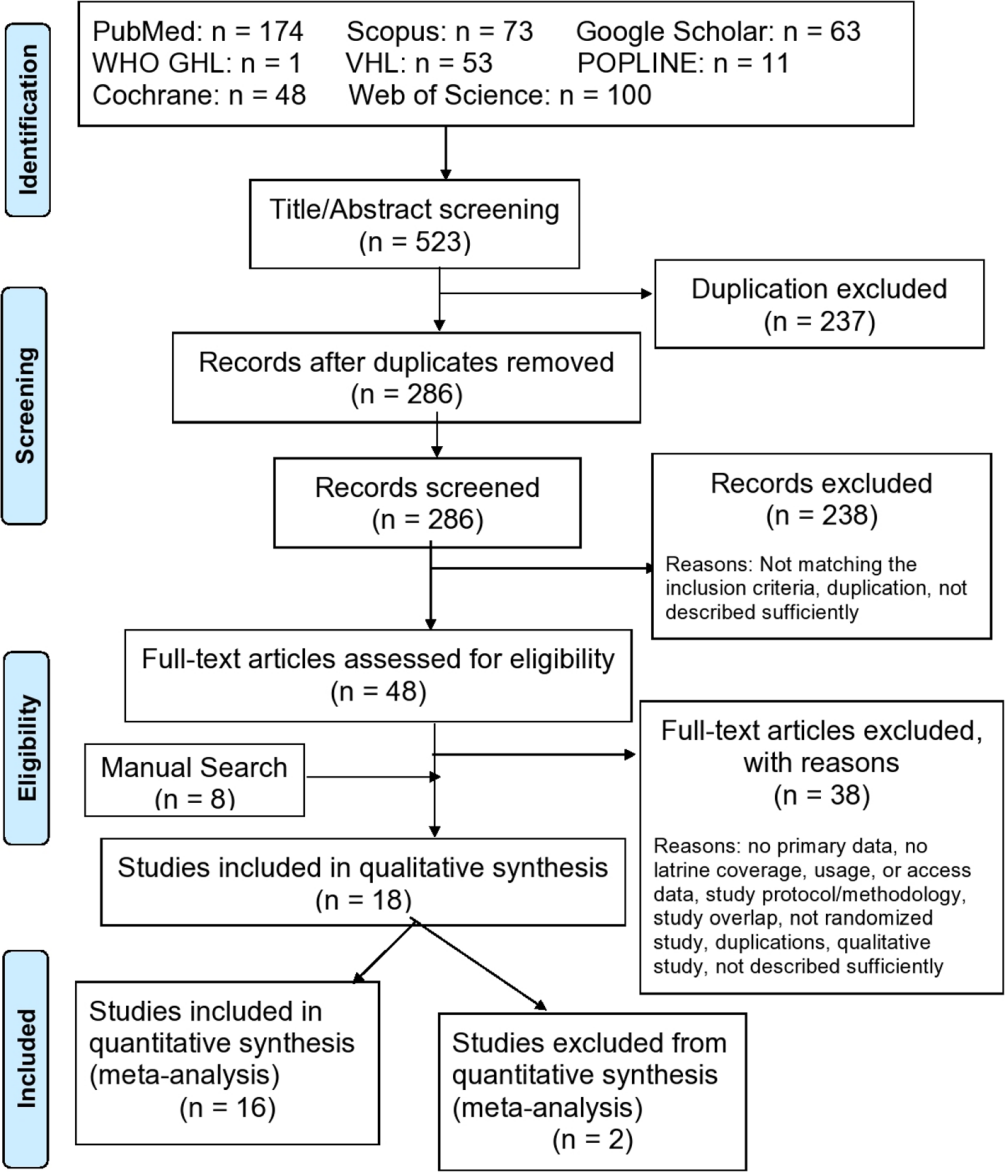
Flow chart of the search and review procedure for the systematic review

The 8 electronic databases searches identified 523 papers. After initial screening, 237 titles were excluded as duplicates. The remaining 286 titles and abstracts were screened for evaluation of inclusion criteria. Two hundred thirty-eight papers were taken out due to lack of eligibility. Afterward, 48 papers were moved further screening in full-text by manually searching for the included articles; eight additional papers were identified. In full-text screening, 38 papers were excluded. Reasons for exclusion included no data on latrine usage, coverage or access, and safe feces disposal, lack of information on study protocol/methodology, not a randomized study, duplications, qualitative study, and not described sufficiently. Finally, 18 studies matched all criteria and were intended to be included in the meta-analysis. However, two studies were not included in the meta-analysis due to inadequate reporting of a measure of variation. Thus, 16 studies were included in the final meta-analysis (Fig. 1).

### Characteristic of the included literature

The 16 included papers were derived from only three regions, Sub-Saharan Africa (7), Southern Asia (8), and Southeast Asia (1) (Table 1). Four studies each were conducted in Bangladesh and India. Two studies were conducted in Kenya. The remaining six papers were from different countries including Burkina Faso, Ethiopia, Mali, Nigeria, Tanzania, and Indonesia. CLTS was launched in Bangladesh and TSC was employed by the government of India.

**Table 1:** Characteristic of included in meta-analysis

Author/Year	Country	Analysis Outcome	Measurement unit	Target population	Intervention time frame
Stanton/1987 [15]	Bangladesh	Safe feces disposal	Community	Family lives in impoverished area. The family has (a) child(ren) at least one child aged under six years	1984-1985
Parvez/2018 [16]	Bangladesh	1) Safe feces disposal 2) Latrine usage 3) Latrine coverage or access	Household	Pregnant women in their second and third trimester	2012-2014
Huda/2012 [17]	Bangladesh	1) Safe feces disposal 2) Latrine coverage or access 3) Improved latrine coverage or access	Household	Household with at least 1 child under 24 months of age	2009
Guiteras/2015 [18]	Bangladesh	1) Safe feces disposal 2) Latrine coverage or access 3) Improved latrine coverage or access	Household	Households with children under the age of 2 years	2012
Erismann/2017 [19]	Burkina Faso	Latrine usage	School	Children age 0-9 years	2015
Stoller/2011 [20]	Ethiopia	1) Latrine usage 2) Latrine coverage or access	Household	Household in Tanore district Rajashi division, In northwest Bangladesh	Not reported
Freeman/2016 [21]	India	Safe feces disposal	Household	Walking ability of their youngest child under 5 years old.	2010-2013
Clasen/2014 [22]	India	Latrine coverage or access	Household	Eligible if they had a child younger than 4 years or a pregnant woman	2011-2012
Patil/2014 [23]	India	1) Safe feces disposal 2) Latrine usage 3) Latrine coverage or access 4) Improved latrine coverage or access 5) Diarrhea	Household	Household with at least 1 child under 24 months of age	Not reported
Pattanayak/2009 [24]	India	Latrine coverage or access	Household	Children aged 0 to 9 years	2006
Cameron/2013 [25]	Indonesia	Safe feces disposal	Household	Households have children under 5 years old	2008-2011
Null/2018 [26]	Kenya	1) Safe feces disposal 2) Improved latrine coverage or access 3) Diarrhea	Household	Household which women were eligible to participate if they reported that they were in their second or third trimester of pregnancy, planned to continue to live at their current residence for the next 2 years,	2012-2014
Caruso/2014 [27]	Kenya	Latrine usage	School	Gread 1-8 students	2010
Pickering/2015 [28]	Mali	1) Safe feces disposal 2) Latrine coverage or access	Household	Households a child at least younger than 10 years	Not reported
Jinadu/2007 [29]	Nigeria	Safe feces disposal	Community	Women with children between 0 and 5 years old	Not reported
Briceno/2017 [30]	Tanzania	Safe feces disposal	Village	Household 1) had been living in the village since the beginning of 2009 or earlier, 2) had at least one child under five years old.	2009-2011

Fourteen out of 16 included papers were published after 2007 (Table 1). The intervention time frame was dependent on the study. The included studies mainly examined community-based interventions. Only two studies were conducted in schools (Table 1). Several studies defined referring to JMP sanitation facility’s definition [4]. For the studies that did not mention whether JMP criteria were applied, it was judged by the included tables and figure captions. Thus, this review was divided into four sanitation outcomes: (1) safe feces disposal; no open defecation, chamber use in children, suitable defecation, (2) latrine usage (determined based on several evidences such as smell of feces, wet pan except when rainy, stain from feces or urine, presence of soap, presence of water bucket or can, presence of a broom or brush for cleaning, or presence of slippers), (3) latrine coverage or access (defined as a households with access to latrine or with connections to sewerage); and (4) improved latrine coverage or access (based on JMP improved latrine definition, it is defined as a potential sanitation system that could be used for separate human excreta at all steps of the sanitation service chain and categorized as flush or pour-flush connected to piped sewer systems, septic tanks or pit latrines, ventilated improved pit latrines, composting toilets, and pit latrine with slabs which are able to separate excreta from human contact). In addition, prevalence of diarrhea was assessed as the secondary outcome.

### Analysis of outcomes in included studies

Each outcome was analyzed and shown in forest plot figures by a random-effects model. Two studies [31, 32] were eligible but could not be included in the meta-analysis due to inadequate reporting of a measure of variation. These data were used for supplement explanation in each outcome arm.

### Safe feces disposal

**Fig. 2:**
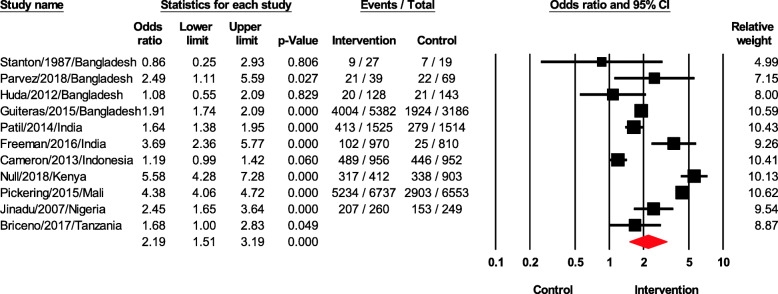
Forest plot of safe feces disposal

The effect of sanitation interventions on safe feces disposal showed a significant improvement with pooled odds ratios (OR) = 2.19 (95% CI 1.51–3.19, *p* < 0.05) (Fig. 2). There was a high heterogeneity between studies (*I*^2^ = 97.28); most of studies except two studies [15, 17] showed significant improvement of interventions compared to the control.

**Fig. 3:**
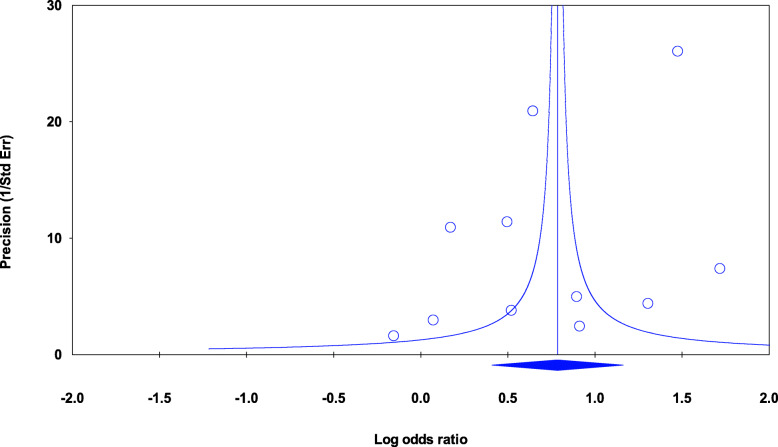
Assessment of publication bias using Begg’s funnel plot

The sensitivity analysis by removing one study showed similar ORs, suggesting that the result was not dependent on any single study. Our Egger’s regression test revealed no publication bias was detected (Fig. 3).

### Latrine usage

**Fig. 4:**
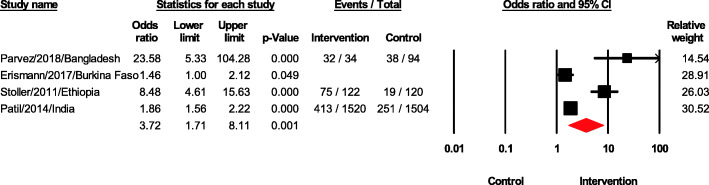
Forest plot of latrine usage

The effects of sanitation interventions on latrine usage are shown in Fig. 4. Data from four papers revealed an overall odds ratio of 3.72 (95% CI 1.71–8.11, *p* < 0.05). Parvez [16] reported an odds ratio of 23.58 (95% CI 5.33–104.28, *p* < 0.05) with a very wide 95% CI compared with other studies. Our sensitive analysis using the one-study remove approach showed the overall odds ratio unchanged. According to Crocker [32], natural leader education contributed to use of private latrine. This corresponded to a larger increase in latrine use of 18.3%.

### Latrine coverage or access

**Fig. 5:**
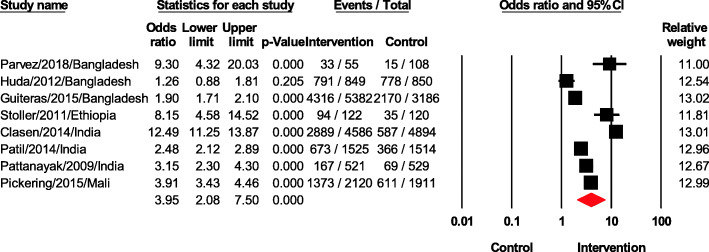
Forest plot of latrine coverage or access

Sanitation intervention had a significant impact on latrine coverage or access, with an odds ratio of 3.95 (95% CI 2.08–7.50, *p* < 0.05). One study [17] conducted in Bangladesh did not show a significant impact of the intervention on latrine coverage or access. The other seven included studies showed significant improvements of interventions on latrine coverage or access regardless of the intervention type (Fig. 5).

**Fig. 6:**
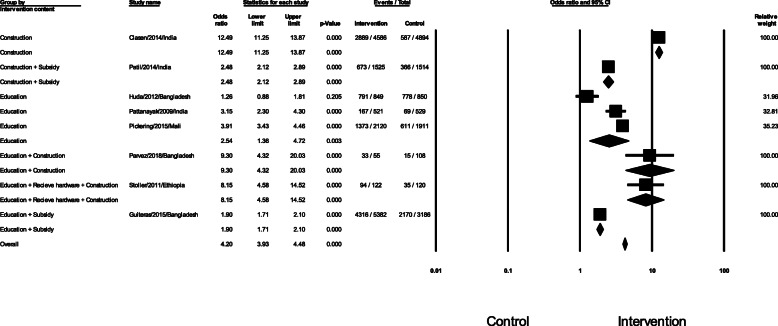
Forest plot of latrine coverage or access group by intervention content

We further assessed the effects of six different intervention types on latrine coverage or access (Fig. 6). Indeed, Clasen et al. performed a randomized *controlled* trial with the intervention consisting of latrine promotion and construction [22]. Additionally, Patil et al. evaluated the effect of the total sanitation campaign *including* the subsidies for and promotion of individual household latrines that can safely confine feces [23]. Regarding education, Huda et al. assessed the SHEWA-B intervention that trained the behavior change communication materials related to water, sanitation, and hygiene of more than 10,000 local residents [17]. Similarly, Pattanayak et al. have developed the information, education and communication (IEC) activities to improve attitudes and knowledge about how sanitation, safe water, and hygiene relate to health for 1050 households in a rural district of India [24]. In the same manner, Pickering launched the community-led total sanitation which aim to change the behaviour of people as eliminate the practice of open defecation in rural communities and promote building of toilets [28]. On the other hand, Parvez et al. *executed* the WASH intervention including education and intervention of water, sanitation, hygiene, and nutrition [16]. Particularly, Stoller et al. reported a cluster-randomized trial evaluating the effectiveness of latrine promotion on chlamydia infection after mass treatment with antibiotics and sanitation education [20]. Finally, Guiteras et al. performed the Latrine Promotion Program with motivation and health information combined with subsidies for the purchase of hygienic latrines [18]. Only education interventions were assessed by several studies. The odds ratio for these studies was 2.54 (95% CI 1.36–4.72, *p* < 0.05). Most of studies using latrine construction with or without education revealed higher odd ratios compared to education alone.

### Improved latrine coverage or access

**Fig. 7:**
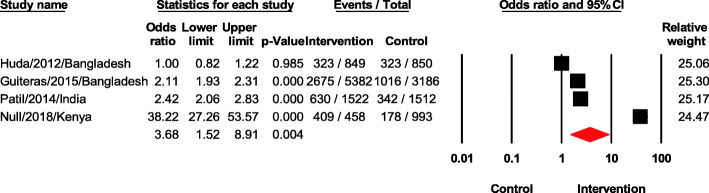
Forest plot of improved latrine coverage or access

Four studies assessed the effects of interventions on improved latrine coverage (Fig. 7). The overall odds ratio was 3.68 (95% CI 1.52–8.91, *p* < 0.05). Heterogeneity was extremely high (*I*^2^ = 99.11). The sensitivity analysis by removing one study did not affect the pooled result. One study in Bangladesh [17] showed no significant effect of the intervention on improved latrine coverage with an odds ratio of 1.00 (95% CI 0.82–1.22, *p* = 0.985).

Biran study [31] that was not included in the meta-analysis assessed improved latrine access at the household including a disabled person. Improved latrine access decreased in both the intervention and control arms during follow-up. Both arms received intervention CLTS. The control group received standard CLTS, and the intervention group received inclusive of disabled people CLTS. Improved latrine access in the household decreased from 19.6 to 14.0% and 29.5 to 20.5% from baseline to follow-up in the intervention and control groups, respectively.

### Diarrhea prevalence

We included papers which reported the prevalence of diarrhea as a secondary outcome. Two studies reported this outcome in the full-text article assessment. However, it was not possible to combine their results due to difference in the content of intervention of each study. Indeed, Patil et al. focused on total sanitation on the defecation behaviors and child health [23], whereas Null et al. assessed a combination of water quality, sanitation, handwashing, and nutritional interventions on diarrhea and child growth [26]. Moreover, these two authors found that there was no association between these interventions and diarrhea prevalence.

### Risk of bias assessment in the included studies

**Fig. 8:**
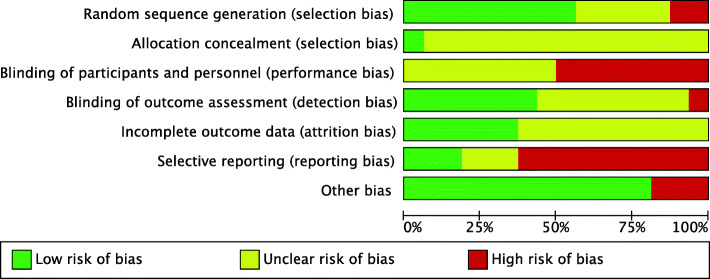
Characteristics of risk of bias

The quality of the included studies was assessed by using the Cochrane risk of bias tool and was represented in Fig. 8 and Supplemental Figure 1. The overall risk of bias ranged in three categories from high, unclear, to low. The highest risk of bias was reporting bias and performance bias. Blinding of participants and personnel was not possible in most of the studies. Reporting bias was common. In terms of sanitation outcomes, latrine usage was measured throughout the household interviews, while latrine existence was more likely to be observed directly by enumerators. This could reduce reporting bias. Detection bias was difficult to evaluate as it was unclear whether enumerators were blinded or not. Attrition bias was not recognized in most papers. According to selection bias in random sequence generation, Briceño and Jinadu were categorized the high risk of bias group [29, 30]. Briceño [30] were applied to select the 190 of 230 largest wards by population size, and Jinadu [29] randomly selected every third household for the study. Both of their selections were not adequately randomized. However, Clasen, Huda, Null, Pickering, Stanton and Clemens, Stoller mentioned that they employed a random-number table or a computerized random number generator [15, 17, 20, 22]. They were categorized at low risk of bias. Another selection bias in allocation concealment was not easily assessed due to less report. In this systematic review, Stoller mentioned the trial arms were concealed from the field teams until the start of the different interventions. Consequently, it was given a low risk of bias [29]. Other studies were presented an unclear risk of bias.

## Discussion

This systematic review and meta-analysis showed that educational interventions improved safe feces disposal, latrine coverage, and latrine approach. Education plus latrine construction also encouraged safe feces disposal (Fig. 6). Our study complements a similar study by Garn [6]. The impact of education intervention on latrine coverage or access was significant (OR 2.54, 95% CI 1.36–4.72) in this study, compared with Garn’s [6] result of 14% (95% CI 3–26) increase in latrine coverage following education interventions [6]. Additionally, Garn [6] found that average household latrine use was 18% (95% CI – 6 to 41) for sanitation education intervention, but this was not statistically significant. There has been another systematic review on the topic [8]; however, it did not involve a meta-analysis.

It is essential to avoid open defecation to prevent exposure; however, Garn [6] did not evaluate this issue. In a study in Indonesia, Cameron [25] found that 54% of households had at least one member who defecates outside, even in villages close to the river [25]. Another study had similar findings suggesting that this was because the latrine was in an inconvenient location [33]. Bhatt found that open defecation was a regular habit in Nepal and people had never felt the need for an alternative [34]. Anuradha found that 91 of 275 (33.1%) study participants in India defecated in the open because there was no latrine at home. The reasons given by the participants for having no latrine were lack of money, lack of space, and/or lack of interest [35]. The findings of this systematic review and meta-analysis suggest that these problems could be solved by using subsidies, providing education, and building latrines. Guiteras found that both latrine construction and subsidies improved latrine use among neighbors [18]. Freeman found that schools in Kenya that received new latrines in a hygiene promotion program were able to reduce their pupil-to-latrine ratio from 77:1 to 41:1, and water treatment and sanitation interventions in schools with water available reduced the ratio from 61:1 to 51:1 compared with the control, increasing access to latrines for pupils [21]. Latrine construction alone probably increases exposure to pathogens through defecation [27, 36]. Therefore, *it* needs to be combined with education in schools and communities [37]. The social, economic, or physiological implications should also be included in interventions [38]. The findings of this study should be used to solve existing sanitation problems and issues, such as achieving communities that are free from open defecation. Sanitation interventions will be necessary to deliver educational behavior change approaches and should be implemented as a total sanitation approach to target safe feces disposal explicitly. Safe feces disposal and use of latrines both improve sanitation, reducing the risk of waterborne infections such as diarrhea and contribute to increased human dignity, privacy, and safety. Sanitation improvement interventions are therefore necessary to improve quality of life as well as to achieve Sustainable Development (Goal 6).

This systematic review and meta-analysis had several limitations. The studies included were from 11 lower- and middle-income countries. Non-English key words were not explicitly included in the search, so studies from some countries may have been missed. For the systematic review, 8 of the 16 studies were from Bangladesh and India, so there was little diversity of geographical characteristics and differences. Two subgroup analyses were performed; the first on education interventions to measure their effectiveness in increasing safe feces disposal, and the second on latrine coverage or access. It is therefore possible that this systematic review and meta-analysis did not reveal the full effect of sanitation interventions. The assessment of risk of bias showed there was a moderate risk of bias. None of the selected studies was defined as low risk, because the sanitation intervention in each case was not completely blinded for the participants. Latrine existence was observed directly by an enumerator and latrine use was determined through structured questionnaires. In some studies, participants were allocated randomly into intervention or control. It was therefore impossible to gauge whether a study was concealed or not. As a result, most of the studies included were considered to have an unclear risk of bias.

## Conclusion

Our study provided the evidence of community- and school-based sanitation interventions in improving latrine use and coverage along with the increase in number of latrine constructions. This study also provided information about effectiveness of sanitation interventions on safe fecal disposal which in turn reduces the incidence/prevalence of diarrheal diseases in the communities. From our evidence-based results, it would be helpful for concerned authorities of any developing countries on implementation of plans for improving the sanitation and reduction of diarrheal diseases.

## Supplementary Information

**Additional file 1: Supplemental Table 1**. PRISMA checklist

**Additional file 2: Supplemental Table 2**. Search terms for each database

**Additional file 3: Supplemental Figure 1**. Summary of risk of bias

## Data Availability

The datasets used and/or analyzed during the current study are available from the corresponding author on reasonable request. Funding: not applicable
