# Supplementary material for: Effectiveness of community and school-based sanitation interventions in improving latrine coverage: a systematic review and meta-analysis of randomized controlled interventions
Source: Environ Health Prev Med. 2021 Feb 24;26:26. doi: 10.1186/s12199-021-00934-4 (PMC7903680; doi:10.1186/s12199-021-00934-4)
Supplement: Supplementary file 2 — Additional file 2: Supplemental Table 2. Search terms for each database [file 12199_2021_934_MOESM2_ESM.docx]

Supplemental Table 2: Search terms for each database

| Databases | Search terms |
| --- | --- |
| Pubmed | (community or communities or school or schools) and (intervention or interventions or interventional) and (sanitation or sanitary or hygiene or hygienic or decontamination) and (randomized or randomised or random or randomly or randomization or randomisation or RCT or RCTs) and (latrine or defecation or defaecation or latrine coverage or latrine usage) |
| Scopus | (community or communities or school or schools) and (intervention or interventions or interventional) and (sanitation or hygiene or sanitary or hygienic or decontamination) and (randomized or randomised or random or randomly or randomization or randomisation or RCT or RCTs) and (latrine or defecation or defecate) |
| Google Scholar | in title “sanitation” or (latrine or defecation or decontamination or sanitary or latrine usage or latrine coverage) |
|  | in title “randomized” or (latrine or defecation or decontamination or sanitary or latrine coverage or latrine usage) |
|  | in title “hygiene” or (latrine or defecation or decontamination or sanitary or latrine coverage or latrine usage) |
| WHO Global Health Library (GHL) | (community or communities or society) and (intervention or interventional or intervene) and (latrine or defecation) and (random or randomized) |
| Virtual Health Library (VHL) | (intervention) and (sanitation or hygiene or sanitary or hygiene) and (latrine or defecate or defecation) and (community or school) and (randomized or randomization) |
| POPLINE | (community OR communities OR school OR schools OR society) AND (intervention OR interventions OR interventional OR intervene) AND (sanitation OR hygiene OR hygienics OR decontamination) AND (latrine OR defecation OR defaecation) AND (randomized OR randomised OR random OR randomly OR randomization OR randomisation OR RCT OR RCTs) |
| Cochrane | (community OR communities OR school OR schools OR society) AND (intervention OR interventions OR interventional OR intervene) AND (sanitation OR hygiene OR hygienics OR decontamination) AND (latrine OR defecation OR defaecation) AND (randomized OR randomised OR random OR randomly OR randomization OR randomisation OR RCT OR RCTs) |
