# Supplementary material for: Effectiveness of community and school-based sanitation interventions in improving latrine coverage: a systematic review and meta-analysis of randomized controlled interventions
Source: Environ Health Prev Med. 2021 Feb 24;26:26. doi: 10.1186/s12199-021-00934-4 (PMC7903680; doi:10.1186/s12199-021-00934-4)
Supplement: Supplementary file 3 — Additional file 3: Supplemental Figure 1. Summary of risk of bias [file 12199_2021_934_MOESM3_ESM.docx]

Supplemental Figure 1: Summary of risk of bias
